# The Ratio 1660/1690 cm^−1^ Measured by Infrared Microspectroscopy Is Not Specific of Enzymatic Collagen Cross-Links in Bone Tissue

**DOI:** 10.1371/journal.pone.0028736

**Published:** 2011-12-14

**Authors:** Delphine Farlay, Marie-Eve Duclos, Evelyne Gineyts, Cindy Bertholon, Stéphanie Viguet-Carrin, Jayakrupakar Nallala, Ganesh D. Sockalingum, Dominique Bertrand, Thierry Roger, Daniel J. Hartmann, Roland Chapurlat, Georges Boivin

**Affiliations:** 1 INSERM, UMR 1033, Lyon, France; 2 Université de Lyon, Lyon, France; 3 MéDIAN, Université de Reims Champagne-Ardenne, CNRS UMR6237-MEDyC, Reims, France; 4 INRA, Nantes, France; 5 UPSP n° 2011-03-101, VetAgro Sup Lyon, Lyon, France; 6 UMR-CNRS 5510, Université Claude Bernard, Lyon, France; University of Liverpool, United Kingdom

## Abstract

In postmenopausal osteoporosis, an impairment in enzymatic cross-links (ECL) occurs, leading in part to a decline in bone biomechanical properties. Biochemical methods by high performance liquid chromatography (HPLC) are currently used to measure ECL. Another method has been proposed, by Fourier Transform InfraRed Imaging (FTIRI), to measure a mature PYD/immature DHLNL cross-links ratio, using the 1660/1690 cm^−1^ area ratio in the amide I band. However, in bone, the amide I band composition is complex (collagens, non-collagenous proteins, water vibrations) and the 1660/1690 cm^−1^ by FTIRI has never been directly correlated with the PYD/DHLNL by HPLC. A study design using lathyritic rats, characterized by a decrease in the formation of ECL due to the inhibition of lysyl oxidase, was used in order to determine the evolution of 1660/1690 cm^−1^ by FTIR Microspectroscopy in bone tissue and compare to the ECL quantified by HPLC. The actual amount of ECL was quantified by HPLC on cortical bone from control and lathyritic rats. The lathyritic group exhibited a decrease of 78% of pyridinoline content compared to the control group. The 1660/1690 cm^−1^ area ratio was increased within center bone compared to inner bone, and this was also correlated with an increase in both mineral maturity and mineralization index. However, no difference in the 1660/1690 cm^−1^ ratio was found between control and lathyritic rats. Those results were confirmed by principal component analysis performed on multispectral infrared images. In bovine bone, in which PYD was physically destructed by UV-photolysis, the PYD/DHLNL (measured by HPLC) was strongly decreased, whereas the 1660/1690 cm^−1^ was unmodified. In conclusion, the 1660/1690 cm^−1^ is not related to the PYD/DHLNL ratio, but increased with age of bone mineral, suggesting that a modification of this ratio could be mainly due to a modification of the collagen secondary structure related to the mineralization process.

## Introduction

With age and in postmenopausal osteoporosis, an impairment in collagen enzymatic cross-links (ECL) occurs, leading to a decrease in bone biomechanical properties [1]–[3]. Enzymatic cross-linking formation in bone collagen is a system of inherent covalent cross-linking, with intermolecular and interfibrillar cross-links which stabilize collagen fibrils. The conventional technique to measure pyridinium ECL is the high-pressure liquid chromatography (HPLC) method, which is based on acid hydrolysis of bone, with several milligrams of bone powder [4]. The quantification of ECL is now possible *in situ* by micro-coring methods, measuring separately osteonal and interstitial concentrations of ECL [5], or by ultrahigh-performance liquid chromatography (UPLC) [6]. Another technique (indirect) for measuring collagen ECL *in situ* has been proposed to analyze “collagen maturity” and especially “PYD/DHLNL”, by Fourier Transform InfraRed spectroscopy Imaging (FTIRI) [7]. It has been proposed, by one research group, that the ratio of two bands in the amide I region (1660/1690 cm^−1^), measured by FTIRI, was linked to ECL maturity, and especially expressed as the ratio of mature PYD/immature DHLNL [7]. However, the band amide I, related to the C = O vibration at 80%, is very sensitive to secondary structure of proteins, and in bone, is highly complex (with type I collagen, non-collagenous proteins, water vibration interferences …). The 1660/1690 cm^−1^ ratio has been only verified on synthetic peptides but has never been, in bone, directly validated and correlated with the PYD/DHLNL measured by HPLC.

A study design using lathyritic rats characterized by a decrease in the formation of ECL was used in order to assess the evolution of the 1660/1690 cm^−1^ ratio by Fourier Transform InfraRed Microspectroscopy (FTIRM) and ECL measured by HPLC. Osteolathyrism in rats is a model to study the deficiency in cross-links content, by controlling the amount of ECL due to the specific inhibition of lysyl oxidase (LOX). LOX catalyzes the oxidative deamination of lysyl or hydroxylysyl residues [8], creating divalent immature cross-links called dehydro-dihydroxylysinonorleucine (DeH-DHLNL), the predominant immature cross-link in bone, and dehydro-hydroxylysinonorleucine (DeH-HLNL) [1], [9]. These divalent immature cross-links further react with another hydroxyallysine to form trivalent mature pyridinium cross-links, such as pyridinoline (PYD), deoxypyridinoline (DPD), or pyrrole [2], [9], [10]. The importance of lysyl oxidase-derived cross-linking was established from animal studies in which lysyl oxidase was inhibited either by nutritional copper-deficiency [11], pyridoxine inhibition [12] or by or the administration of β-aminopropionitrile (BAPN), an inhibitor of lysyl oxidase. This resulted in osteolathyrism, characterized by weaker bone and exostoses [3].

In order to reinforce our study, we also used bovine bones, in which the PYD has been physically destructed by UV-photolysis, whereas immature DHLNL were not affected, and compared with FTIRM measurements.

Thus the aim of this study was to verify if the 1660/1690 cm^−1^ measured by FTIRM was related to PYD/DHLNL measured by HPLC.

## Materials and Methods

### Animals study design

**Figure 1:**
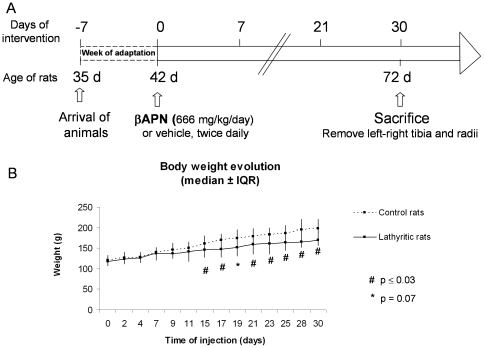
Study design. **A.** Animal study design. **B.** Body weight of control (full line) and lathyritic (dotted line) rats: the body weight in lathyritic rats started to decrease from the 14th day of ßAPN injection.

The study was approved and carried out in accordance with the recommendations of the Ethical Committee of VetAgro Sup (Ethical Committee Guidelines, protocol agreement no. 0702). Twenty virgin female Wistar rats aged of 35 day-old (95 g±5 g) were purchased from Charles River France. There were 4 animals in each cage and they were maintained at 21°C and under a 12∶12-h light/dark cycle at VetAgro Sup (Lyon, France). The animals had free access to tap water and food (standard diet, A04, SAFE) during the entire experiment, and their body weights were monitored every 2 days. At the beginning of the second week, the food was grinded for all the rats. Indeed, as lathyrism also affects alveolar bone, rats had some difficulties to eat. Rats were not pair-fed in this study, as intakes were not expected to be different between groups. After the week of adaptation with a standard diet, rats were randomized according to the body weight into 2 groups: half of the rats in each age group were allocated to be the control groups (CTRL; 120 g±7 g) and the other half were injected with beta-aminopropionitrile (βAPN, 118 g±9 g) to induce the osteolathyrism. The CTRL group was injected with the vehicle only (NaCl 0.9% w/v), and the βAPN group was injected with 666 mg/kg/day of ßAPN (Sigma, Saint-Quentin-Fallavier, France), as reported by Brüel *et al.* [13] Rats were injected twice daily subcutaneously in the nape of the neck during 30 days (Fig. 1A). A veterinarian closely monitored rats for adverse reactions every day. Rats were weighted every 2 days. At necropsy, after 30 days of treatment, animals were anesthetized by intra peritoneal injection of ketamine (70 mg/kg, Imalgene 1000®, Merial Lyon, France) and xylazine (10 mg/kg, Rompun® 2% (v/w); Bayer HealthCare, Gaillard, France), then euthanized by intracardiac injection of sodium pentobarbital euthanasia solution (Dolethal®, Vétoquinol, Lure, France). Bones (radii and tibiae) were separated from adjacent tissue, cleaned, and stored at −20°C for biochemical measurements.

### Rat bone preparation

Right tibiae and radii were frozen at −20°C for biochemical cross-links measurements. Left tibiae and radii were fixed in 70% ethanol for 2 weeks for histology analysis and FTIRM. Then bone samples were dehydrated for 48 h in 100% ethanol, substituted in methylcyclohexane for 48 h and embedded in methylmethacrylate (MMA). For bone histology, undecalcified sections (8 µm-thick) were cut with a microtome Polycut E (Reichert-Jung, Leica, Germany), longitudinally and sections were then stained with Trichrome Goldner. For FTIRM analysis, 2 µm-thick sections were cut and stored between 2 slides.

### Bovine bone slices

150 µm-thick slices of cortical bovine bone of two different ages (2 year-old and 4 year-old) were cut with an Isomet diamond saw. Slides were stored to −20°C until biochemical and FTIR analyses.

### Degradation of pyridinoline by UV photolysis in bovine bone

Four slices were submitted to UV photolysis (365 nm) among the Sakura' procedure [14]. In order to avoid the heating of bone samples, slices were incubated in PBS within Ministat bath at 4°C during all the duration of the experiment. After 72 hrs of UV photolysis, a part of bovine bone slices were used to measure mature and immature cross-links, and the other part were used for FTIRM analysis after ethanol fixation and PMMA embedding.

Four others slices have been submitted to the same protocol, and used for FTIR analysis on bone powder, to verify that the results are not due the ethanol fixation.

### Biochemical analysis: Quantification of mature trivalent and immature divalent enzymatic cross-links of bone by HPLC

The proximal and the distal metaphysis of rat tibiae and radii were removed with a diamond saw and then the diaphysis were cautiously washed with demineralised water to eliminate bone marrow and hydrolyzed by 6 M HCl at 110°C during 20 h. Bone hydrolysates were pre-fractioned by Separation Phase Extraction Chromatography (SPE) on Chromabond® Cross-link Columns (Macherey Nagel GmbH & Co.KG, Düren, Germany), as described previously [4]. Then ECL separation was performed by HPLC on a C18 Atlantis® T3 reversed-phase column with heptafluorobutyric acid as ion-pairing reagent in an acetonitrile-water mobile phase. ECL were monitored for fluorescence at an emission of 395 nm and an excitation of 297 nm (multi-λ fluorescence detector, Waters 2475), and quantified against a calibrator supplied by Quidel Corporation (San Diego, CA, USA). The amount of PYD, DPD and collagen were determined by hydroxyproline HPLC assay (Biorad, Munchen, Germany). Collagen content was calculated assuming 14% hydroxyproline in type I collagen. The resulting data were then used to calculate the cross-link values as mol/mol of collagen.

Concerning bovine bone, biochemical assays of mature and immature cross-links were performed on bovine bone, as described previously [15]. Bones were cut in small pieces and were first demineralised in 0.5 M EDTA in 0.05 M Tris buffer, pH 7.4 for 96 h at 4°C. After extensively washing with deionized water, the suspension was reduced with NaBH_4_ at room temperature before acid hydrolysis in 6 M hydrochloric acid at 110°C for 24 h. Bone hydrolysates were then prefractioned on SPE Chromabond® Cross-link Columns. The cross-links were separated on a C18 Atlantis® T3 reversed-phase column as described before and the detection was carried out by electrospray ionization mass spectrometry in a positive ion mode with selected ion recording [15].

### FTIRM and FTIR analyses

FTIRM was performed on 2 µm-thick sections from rat cortical bones. Bone sections were maintained between 2 sample holders with specific diameter holes. Each spectrum was collected at 4 cm^−1^ resolution and 50 scans in the transmission mode and analyzed with a Perkin-Elmer GXII Auto-image Microscope (Norwalk, CT, USA) equipped with a wide band detector (mercury-cadmium-telluride) (7800-400 cm^−1^). The instrument used an objective Cassegrain of numerical aperture 0.6; the system has a spatial resolution of 10 µm at typical mid-infrared wavelengths. For each sample, 20 measurements were done at 35×35 µm of spatial resolution. Contribution of air and MMA were subtracted from original spectrum. The MMA subtraction was performed by nullifying the main peak of MMA at 1730 cm^−1^ (carbonyl absorption band) An automatic baseline correction (quadratic function) was done on spectra with the Spectrum Software, and the curve fitting of every individual spectrum was performed.

For bovine bone, one part of both control and submitted to UV photolysis, was performed on KBr pellets containing 1% (wt/wt) of collagen powder, and the other part was fixed in ethanol 70% and embedded in PMMA, in order to analyze the effect of fixation on the 1660/1690 cm^−1^ area ratio.

GRAMS/AI software (Thermo Galactic, Salem, NH, USA) was used to quantify the characteristics of the spectra. After peak-fitting deconvolution of every individual spectrum, position, height, width at half height and area under the curves were obtained. Peaks corresponding to amides I (1600–1700 cm^−1^), ν_1_ν_3_PO_4_ (900–1200 cm^−1^), were analyzed. The Amide I vibration was curve-fitted into 3 mains components, 1690 cm^−1^, 1660 cm^−1^ and 1633 cm^−1^ [16]. These 3 peaks frequencies were fixed during the fitting, and 6 others peaks corresponding to the others amide vibrations were added. For the amide vibration, a total of 9 peaks were fitted altogether. The following variables were calculated: (1) mineral maturity (1030/1110 cm^−1^ area ratio)[17], (2) mineralization index (1184-910 cm^−1^/1712-1592 cm^−1^)[18], (3) collagen maturity (1660/1690 cm^−1^ area ratio) [7].

### FTIR Imaging and Multivariate data analysis

Infrared images on cortical bone were recorded on a Spotlight 300 microscope coupled to a Spectrum One spectrometer (Perkin-Elmer, Courtaboeuf, France) equipped with a liquid nitrogen cooled 16-element MCT detector. Each pixel element had a size of 6.25 µm. Spectra were recorded in the 4000-720 cm^−1^ range, at 4 cm^−1^ spectral resolution, and using 16 scans per pixel. The mean acquisition time was about 6 hrs per image. Five control and 5 lathyritic radii were recorded and resulting infrared images were analyzed by multivariate data analysis.

Several steps were used before extracting information from the IR images. A multivariate data analysis was carried out on whole spectra from IR images by using MATLAB (The Mathworks inc., Natik MA, USA). Five control and 5 lathyritic radii images were analyzed in this study.

#### Random sampling

Each IR image containing more than 26.10^3^ spectra, the whole data set has been reduced before the analysis process. A sampling was performed on infrared images by randomly selecting 1000 spectra/image. The spectral window was 2000-750 cm^−1^ range, giving 1626 data points. Finally, all selected spectra were gathered in a single data matrix, with a total of 10 IR images, thus with 10000 rows, corresponding to 10 images and 1000 spectra per image, and 1626 data points (wavenumbers).

#### Thresholding

In each image, the spectra were taken as supplementary observation of the previous principal component analysis (PCA). In order to automatically identify the bone-region from the background region, it was necessary to define a threshold, which attributed the pixel to one of these two groups. In this way, the spectra were projected in a reduced space, more convenient for data analysis. After mathematical subtraction of MMA peak and baseline correction, IR images were processed by thresholding in order to detect the bone region from the background. To the end, transformations of IR images into a very low-dimensional space were performed with a PCA, in order to visualize high-dimensional data. The visual examination of the first score-image showed us that the background was associated with negative scores, whereas the positive scores were associated to the bone regions.

#### Image labelling

In this approach, previously described in *Bonnier et al.* [19], the spectra of the “bones” regions of both lathyritic and normal rats were grouped using an unsupervised clustering approach based on the KC mean algorithm, with 10 groups to be identified. Each pixel spectrum was thus classified in one of these 10 groups. A matrix G with 10 rows (rats) and 10 columns (identified group) was built up from the clustering. In this matrix, an element *gij* gives the proportion of the pixels from the image *i*, which have been classified in the group numbered *j*. The matrix G was again processed by ANOVA and other statistical methods. Both 1660/1690 cm^−1^ absorbance ratio and 1030/1110 cm^−1^ absorbance ratio were expressed in false colors on images of both control and lathyritic radii. The background is set to 0, and the maximum is in red. The 1030/1110 cm^−1^ and 1660/1690 cm^−1^ absorbance ratios in false colors were calculated from images of control bone and lathyritic bone.

### Statistical analyses

To test the differences between normal and lathyritic animals, a non-parametric Mann Whitney U Test was used. Spearman correlation was used to test correlation between the 1660/1690 cm^−1^ area ratio (collagen maturity) and the 1030/1110 cm^−1^ area ratio (mineral maturity). Data are presented as median ± Inter Quartile Range (IQR) unless otherwise noted. The level of significance was set at p<0.05 for all statistical tests. All statistical analyses were performed using the StatView software.

## Results

### Clinical findings in osteolathyritic rats

Compared to controls rats, lathyritic rats exhibit typical signs of osteolathyrism, as a decrease of activity and a failure to gain weight, 14 days from the beginning of ßAPN injections, compared to controls rats. After the 14th day of treatment and up to 30 days, the body weight was significantly decreased in the ßAPN-treated group (at day 30: 198±21 g vs 169±13 g, −14%, p≤0.03, Fig. 1B) when compared to the control group.

### Histological evaluation

**Figure 2:**
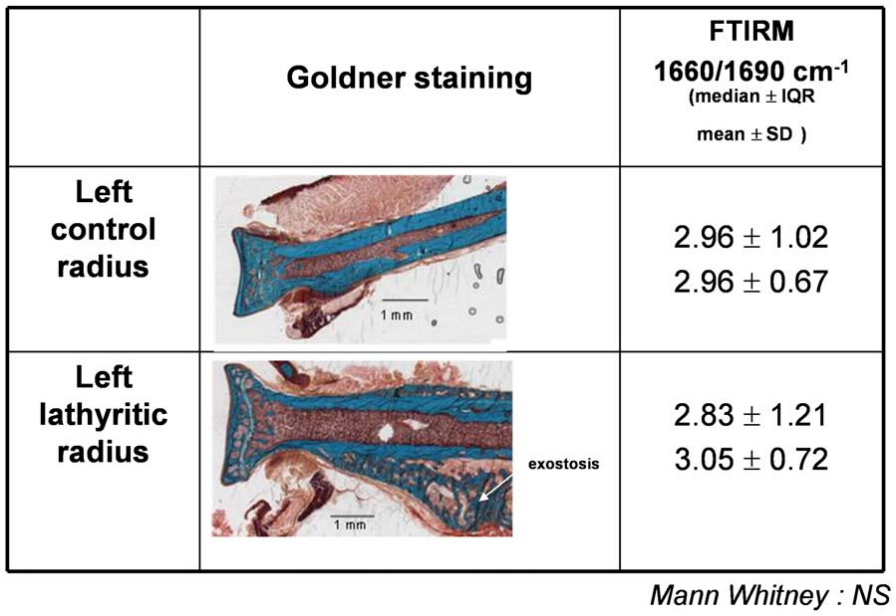
Comparison of histological images showing a major exostosis on lathyritic radius. FTIRM analysis of 1660/1690 cm^−1^ area ratio revealed no significant difference between control and lathyritic bone.

During bone dissection, it has been observed that soft tissues adjacent to bone were much more fragile and easier to remove in lathyritic than in control rats. Blood vessels were easily broken off during dissection. Histological evaluation revealed that in lathyritic bone, exostoses were always present on lateral part of bones, including tibia, radius, femur, humerus, rib (Fig. 2).

### Quantification of trivalent cross-links and collagen contents in rat bones by HPLC

Compared to the control group, a significant decrease in PYD and PYD+DPD contents were found in the lathyritic group, both in the tibiae and radii (Table 1). The decrease of PYD and PYD+DPD content was more important in the radii than in the tibiae (PYD: −78% vs −22%, respectively; PYD+DPD: −64% vs −19% respectively), consequently radii were selected for FTIRM analysis.

**Table 1:** Biochemical dosages of pyridinoline (PYD), and PYD+DPD (D-pyridinoline) in radii and tibiae.

Groups	Control rats(n = 10)	Lathyritic rats (n = 10)	% of difference
	mean ± SD	mean ± SD	
	median ± IQR	median ± IQR	
**Tibia PYD** (mmol/mol coll)	142±16	104±16	−27%*
	140±22	109±30	−22%*
**Tibia PYD + DPD** (mmol/mol coll)	377±51	279±45	−26%*
	366±82	299±78	−19%*
**Radius PYD** (mmol/mol coll)	178±58	43±9	−76%*
	193±58	44±9	−78%*
**Radius PYD + DPD** (mmol/mol coll)	383±39	147±33	−62%*
	378±57	138±42	−64%*

*Mann Whitney, p≤0.0006.

PYD and PYD+DPD contents decreased in lathyritic bones compared to the control bones.

### FTIRM analysis

**Figure 3:**
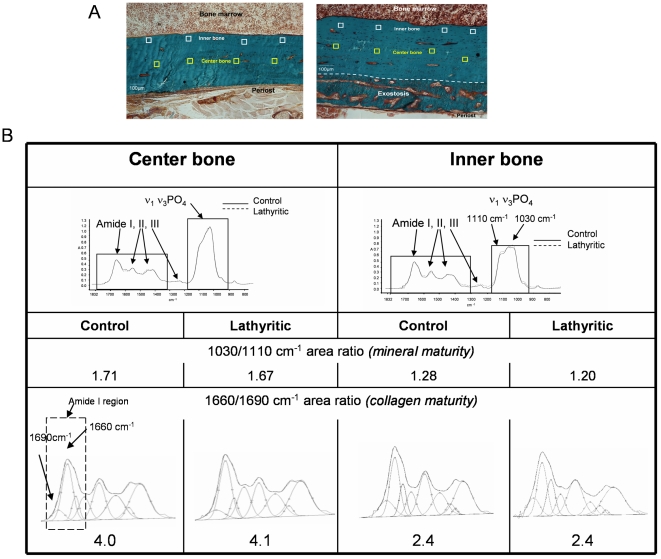
FTIRM analysis. **A.** Sections of bone (radii) from control (left) and lathyritic (right) rat, stained with Trichrome Goldner. □: zones of measurements (35×35 µm) performed by infrared microspectrocopy either in inner bone (near bone marrow) in white, or in central bone in yellow (10 measurements in each cortical, 4 shown). The dash line indicates the exostosis in lathyritic bone, in lateral part of bone. **B.** Infrared spectra of center and inner bone in control and lathyritic bones, with curve fitting of amide I and ν_1_ν_3_PO_4_ vibrations. For a given mineral maturity (1030/1110 cm^−1^ area ratio), there was no difference in the collagen maturity (1660/1690 cm^−1^) between control and lathyritic bones. However, collagen maturity and mineral maturity area ratios were higher in the center bone compared to the inner bone in both control and lathyritic bones.

No significant difference was found in the collagen maturity between the control and lathyritic groups (Fig. 2), even though there was a deep decrease in PYD content on radii (Table 1). However, in cortical bone, collagen maturity in interstitial bone was always greater than in young bone tissue close to bone marrow (inner bone), both in control and lathyritic bone (Fig. 3). This can be easily observed on infrared spectra (Fig. 3); in center bone, v_1_v_3_PO_4_ (mineral vibration) was higher than in inner bone, for a same intensity of amide I (organic matrix), suggesting that the amount of mineral is higher in center than in inner bone. In center bone, for mineral maturity both in control and in lathyritic groups (1.71 vs 1.67, respectively), no difference in collagen maturity could be observed (4.0 vs 4.1). In inner bone, mineral maturity was reduced compared to the center bone, both in control and lathyritic bones (1.28 vs 1.20, respectively), and collagen maturity decreased in parallel (2.4 in both control and lathyritic bones). Thus for a given mineral maturity, collagen maturity did not vary between control or lathyritic bones (Fig. 3).

**Figure 4:**
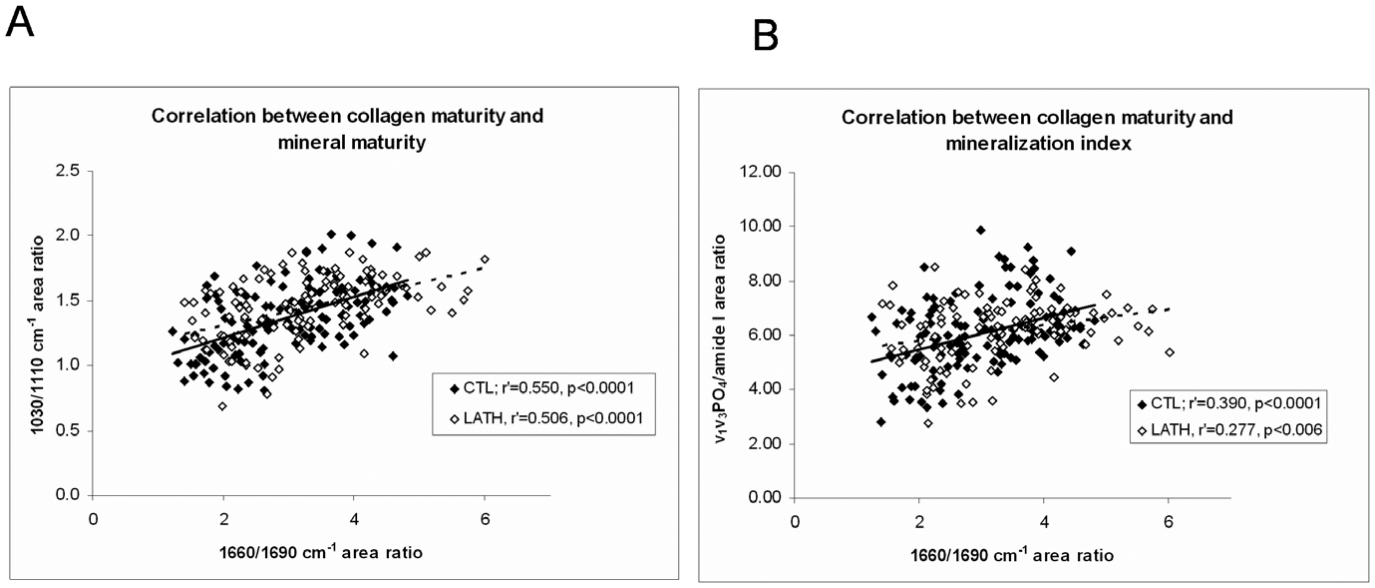
Correlations between collagen and mineral parameters. **A.** Spearman correlations showing that the 1660/1690 cm^−1^ area ratio increased with the mineral maturity of the both in the control or in the lathyritic radii **B.** Spearman correlations showing that the 1660/1690 cm^−1^ area ratio increased with the mineralization index both in the control or in the lathyritic radii.

To examine the relationship between the collagen maturity and the mineralization, correlations between 1660/1690 cm^−1^ (collagen maturity) and 1030/1110 cm^−1^ area ratio (mineral maturity), and collagen maturity and mineralization index were performed, both in control and lathyritic radii. Significant positive correlations were observed in these 2 groups of rats between both collagen maturity and mineral maturity (Fig. 4A), and collagen maturity and mineralization index (Fig. 4B).

### Chemometrical study on bones from control and lathyritic rats

#### Random sampling

In each trial, the spectral data points were considered as response in a one-way ANOVA with the nature “normal/lathyritic” as factor. The profiles of F-Fisher value were compared with the one resulting from several random repartitions of the spectra. It was expected that the F-Fisher values will be larger on “real spectra” collection in comparison with “randomized” spectral ones. Tests were not significant, thus this it cannot be concluded that the spectra from normal bone significantly differed from the lathyritic ones.

#### Thresholding

In a second study, the regions correspond to bone matrix were isolated from the images. For this purpose, PCA was applied on the images and the “bone” region was isolated by thresholding of the score-image related to the first dimension. The regions with the pixel-score greater than 1 were considered as being constitutive of the “bone” region. The spectra from the bone regions of lathyritic rats did not significantly differ from the spectra of the bone region of normal rats.

#### Image labelling

**Figure 5:**
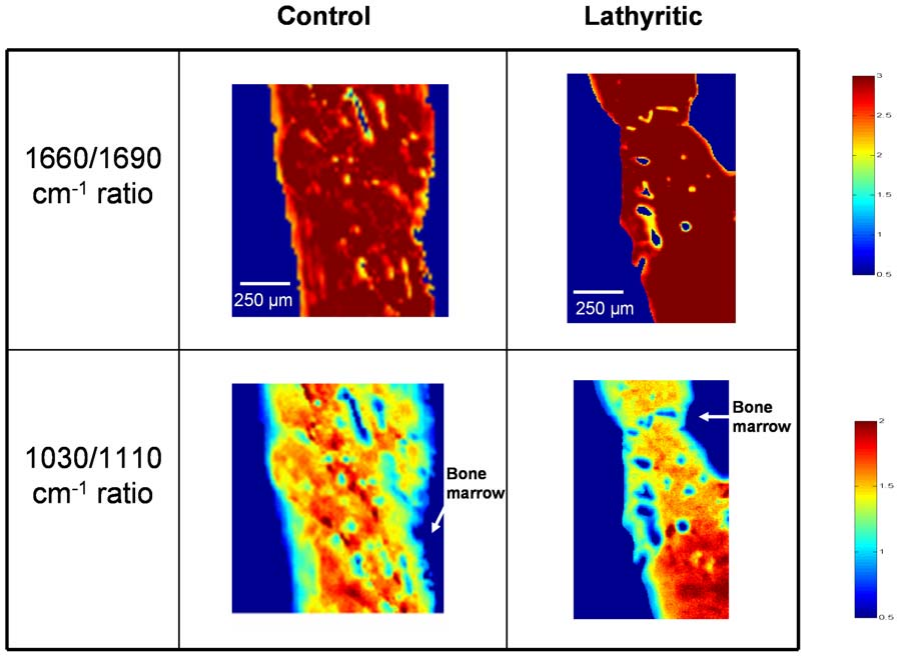
Image representations in false color of 1660/1690 cm^−1^ and 1030/1110 cm^−1^ of a control (left) and a lathyritic radius (right). The background is set to 0 (blue color). No discrimination between the 2 groups (control and lathyritic bones) was observed in the 1660/1690 cm^−1^ ratio. Inside a given bone region, the variations in false color from the boundary to the inside emphasize the differences in the collagen maturity and mineral maturity according to the location.

No proportions of group significantly differed according to the normal/lathyritic nature of the observations. This meant that there was no “spectral signature” which depends on the studied factor. Then, 1660/1690 cm^−1^ and 1030/1110 cm^−1^ absorbance ratios were calculated and represented in false color. The 1660/1690 cm^−1^ absorbance ratio (Fig. 5) showed an increase of the collagen maturity ratio within bone whereas this ratio decreased in edge of bone, confirming that collagen maturity was higher in center bone than in inner bone both in the control and in the lathyritic bones. Similar results were found with the mineral maturity [i.e. 1030/1110 cm^−1^ absorbance ratios (Fig. 5)].

### Effect of UV photolysis on mature and immature crosslink concentration

#### Dosage of mature and immature cross-links before and after UV photolysis

**Figure 6:**
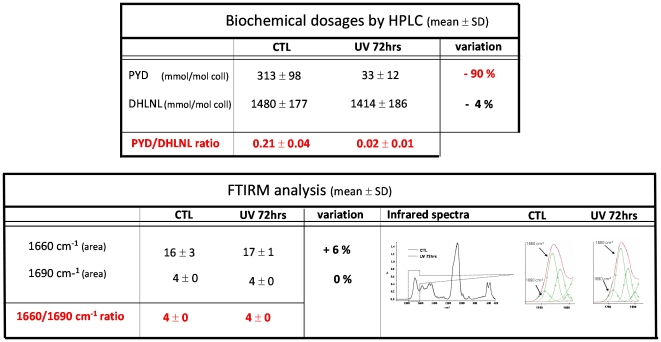
Effect of 72 hrs UV photolysis on enzymatic cross-links on cortical bovine bone measured by HPLC, and on the 1660/1690 cm^−1^ ratio by FTIRM. Despite a high decrease of mature PYD cross-links after UV photolysis (and not immature DHLNL cross-links), no difference in the 1660/1690 cm^−1^ area ratio was observed by FTIRM analysis.

Before UV photolysis, in the 2 yrs bovine bone, the concentration of mature cross links, i.e. PYD and DPD, were 243 and 16 mmol/mol coll, respectively. Concentration of immature cross links, i.e. DHLNL and HLNL, were 1355 and 437 mmol/mol coll, respectively. After 72 hrs of UV photolysis at 4°C, PYD and DPD were 24 and 2 mmol/mol coll, (decreased by ≈90% and 89% respectively), and DHLNL and HLNL were 1283 and 411 mmol/mol coll (−5% and −6% respectively, Fig. 6).

In the 4 yrs bovine bone, the concentration of mature cross links, i.e. PYD and DPD, were 382 and 52 mmol/mol coll, respectively. Concentration of immature cross links, i.e. DHLNL and HLNL, were 1605 and 460 mmol/mol coll, respectively. After 72 hrs of UV photolysis at 4°C, PYD and DPD were 41 and 4 mmol/mol coll, (decreased by ≈89% and 93% respectively), and DHLNL and HLNL were 1546 and 501 mmol/mol coll (−4% and +9% respectively).

#### Effect of destruction of mature crosslink by UV photolysis on 1660/1690 cm^−1^ area ratio measured by FTIR analysis

Despite a huge decrease of PYD and DPD (≈90%), no difference was observed in the 1660/1690 cm^−1^ area ratio. Results were shown for the 2 yrs bovine bone but were exactly the same for the 4 yrs bovine bone (Fig. 6).

Concerning the effect of ethanol fixation and embedding in PMMA, while an overall reduction of the amide and phosphates bands was observed in the ethanol-fixed bone, due to the bone dehydration, no difference were observed between UV photolysis submitted bone and control bone (data not shown).

## Discussion

The present study investigated, in a lathyritic rat model, the relationship between PYD/DHLNL ratio measured biochemically by HPLC, and the 1660/1690 cm^−1^ band ratio proposed by Paschalis et al. [7]. At the beginning of our experiments, only the dosages of mature cross-links PYD and DPD were available in the laboratory. As no difference was observed between control and lathyritic bones by FTIRM in the 1660 cm^−1^ area (attributed to PYD among Paschalis et al.), and despite a huge decrease in PYD and DPD measured by HPLC, we decided to develop (and among other reasons) the dosage of immature cross-links in the laboratory [15], to verify the results obtained on rat bone. Thus, bovine bone, in which we have destructed UV-photolysis by both PYD and DPD, was used. The immature cross-links, without pyridinium ring, were not affected by UV-photolysis. Both PYD and DHLNL were dosed by HPLC and compared to the 1660/1690 cm^−1^ ratio performed by FTIRM. While a high decrease was observed in bone submitted to UV-photolysis in the PYD/DHLNL ratio, measured by HPLC, no change of the 1660/1690 cm^−1^ ratio was found.

Experimental lathyrism has been studied in young animals, especially in rats [20]–[25] and chicks [26], [27]. In the present study, we used female Wistar rats aged 42 days at the beginning of injections to reproduce experimental conditions used in previous studies [13]. In a study in rats, Oxlund *et al.* [3] used a dose of 333 mg/kg/day and obtained a decrease of 45% of PYD content in femur bone. We used the dose of 666 mg/kg/day of BAPN, as reported in the study of Bruël *et al.* [13] because we wanted a stronger LOX inhibition compared to Oxlund *et al.*, while maintaining the animal welfare. In the present study, the final body weight in lathyritic rats was decreased by 14% compared to control rats, and started from the 14th day of injection. This decrease in body weight was previously observed. In the present study, exostoses were observed in several bones, as found in others studies [20], [24], [25]. Exostoses or periosteal new bone formation observed in our rats were probably due to the loss of tensile strength of Sharpey's fibers because removal of muscle attachments or denervation of the limb prevented exostoses [24], [25].

The inhibition of ECL in lathyritic rats was clearly demonstrated in this study, with a significant decrease of PYD and PYD+DPD content compared to the control group. While the ratio 1660/1690 cm^−1^ initially was described for corresponding to PYD/DHLHL, the PYD alone and the PYD+DPD were both measured in the present study to see if a correlation could be found. At sacrifice (72 day-old), in the control group, the PYD content quantified in tibiae was 140 mmol/mol collagen and 193 mmol/mol collagen in radii. The content of PYD was consistent with previously reported values in rat femur from the same age [3], [28]. The higher decrease in PYD and PYD+DPD content in radii than in tibiae might stem from higher mechanical solicitation of forelimbs than hindlimbs [29].

The measurement of the collagen maturity (1660/1690 cm^−1^ area ratio) was performed only in the radii which had the most important decrease in PYD content. The ratio was calculated using peak-fitting analysis, with 3 main peaks in the amide I band as previously published [16]. No difference in the 1660/1690 cm^−1^ area ratio was found between control and lathyritic bones, despite a great difference in PYD and PYD+DPD contents (−78% and −64%) measured by HPLC. When our FTIRM experiments showed no differences between the 1660/1690 cm^−1^ band area ratio in control and lathyritic bones, we have decided to confirm our results in an independent laboratory, by FTIR imaging and chemometric analyses. This laboratory (CNRS UMR6247) is equipped with an FTIR Imaging system and have expertise in both spectroscopy and collagen matrices. A second independent laboratory, expert in chemometric analysis (INRA, Team “Bioinformatique', Nantes, France) analyzed the FTIR images by multivariate statistical analysis on whole raw spectra and without curve-fitting, in order to see if a discrimination between control and lathyritic bones could be detected. Three chemometrical approaches have been attempted to verify if a spectral signature was present in lathyritic bones, and confirmed our results showing that what is observed in a point mode is not artefactual but is present over larger areas. This powerful statistical method is based on the analysis of each wave number and can detect even minor modification between samples. Thus this statistical methodology has not detected discriminations in the 1660/1690 cm^−1^ area ratio between control and lathyritic bone, but this does not mean that any spectral signature do not exist between the 2 groups.

The organic matrix can be studied on amide vibrations, specifically on amide I band (∼1600–1700 cm^−1^) [30], [31]. Amide I band in IR spectroscopy is mainly due to the C = O stretch vibration (80%) of the peptide linkages that constitute the backbone structure and this band has often been used to determine the secondary structure of proteins in solution [32], [33]. In collagen, the amide I band corresponds to the C = O stretching of 300 triplets Gly-X-Y (with X and Y are mainly proline and hydroxyproline). Several studies have shown that the main band of amide I (1660 cm^−1^) results from the interaction between carbonyls groups of proline in X position and amine groups of glycine from another triplet [31], [34]. The 2 others bands (∼1690 and 1630 cm^−1^) are due to interactions with water. Amide I can be curve-fitted into 3 main components, with the main peak at 1660 cm^−1^, and the 2 other shoulders in high (∼1690 cm^−1^) and low (∼1633 cm^−1^) frequencies. Pioneer studies on prediction of secondary structure of proteins by IR spectroscopy have shown the complexity but also the usefulness of the amide I vibration to predict the amount of α-helical (∼1654 cm^−1^), ß-structure (∼1675 cm^−1^,1637 cm^−1^, 1631 cm^−1^, 1624 cm^−1^), random coil (∼1645 cm^−1^), turns and bends (∼1694 cm^−1^, 1688 cm^−1^, 1683 cm^−1^ and 1670 cm^−1^) in proteins [30], [32], [35], [36]. A strong band of water is also present at 1640 cm^−1^ [37]. In bone, there is ∼90% of type I collagen, but also ∼10% of non-collagenous proteins. Two of them are abundant in rat bone tissue, i.e., bone sialoprotein (BSP) and osteocalcin [19] [38], [39]. BSP exhibits a majority (>45%) of random coils but also α-helix [40]–[42], whereas OC has a majority of α-helix [43], [44]. Hence, non-collagenous proteins in bone have also a non-negligible contribution in the amide I vibration interfering with the 1660/1690 cm^−1^ measurement.

While no difference in the 1660/1690 cm^−1^ area ratio was found between control and lathyritic bones, a difference in this ratio was shown depending on the location in the bone, with a higher ratio in center bone (“older bone”) than in bone close to bone marrow (inner, more recent bone). Significant correlation were found between the 1660/1690 cm^−1^ (collagen maturity) and the 1030/1110 cm^−1^ area ratios (mineral maturity) and mineralization index, as shown in previous studies [7], [45]. Such a correlation found both in the control and in the lathyritic rats, suggests that the 1660/1690 cm^−1^ area ratio reflects probably more a modification of secondary structure of collagen linked related to the dehydration of the mineral phase than a modification of the enzymatic cross-link content. It has been shown that during mineralization, the apatite crystals replaced some of the molecules of water so their content was inversely proportional to that of water [46], [47], and that Bragg-spacing of collagen strongly decreases with increasing mineral content [48]. This indicated the close relationship between water and the mineral deposition process and modification of the collagen packing probably influences secondary structure of organic matrix. This could be due to a different packing of triple helices between old and newly formed bone, with a tighter packing and a consequent loss of water as the fibrils matured. Of course, the ECL content also changed with age of bone, with a higher content in interstitial than in osteonal bone [5], [49].

As Yeni et al have showed that the alcohol fixation and embedding media could influence the Raman spectra [50], and to confirm that the absence in 1660 cm^−1^ variation was not due to a effect of ethanol fixation, freeze bovine bones of 2 different age were submitted to UV photolysis which has destructed ≈90% of PYD. No variation in 1660 cm^−1^ peak was observed, despite a once more a great decrease in PYD content. This absence of effect was also observed in both alcohol-fixed and unfixed bone, thus excluding a possible bias of ethanol fixation on our results.

We have several explanations for the discrepancies between Paschalis' study [7] and ours. First, the model used in the Paschalis study was the vitamin B-6 deficient chick model of cross- links deficiency. However, in this condition, it was previously shown that collagen fibers were larger (+2.5 fold) than in controls [51], [52]. Thus this model can not to be used to compare modification on amide I vibration. In the lathyritic rat model, no modification in collagen fibers size was demonstrated. Second, they used a series of cross-linked peptides to validate the attribution of either PYD or DHLNL. However, the amino acids composition was different between the 2 peptides (12 Gly DHLNL –containing peptide, versus 26 Gly in PYD-containing peptide, among others). A different composition in amino acids inevitably influences the amide I vibration [32]. Thirdly, the number of peaks used in the curve fitting process was different between the comparisons of the 2 cross-linked peptides previously mentioned. So, the results can not be compared directly. In our study, we always used 3 peaks in all bone samples. Fourthly, one measurement was performed on both bovine bone of 4 months years-old and 2 years-old, and concluded that the 1660/1690 cm^−1^ ratio was higher in the latter. But, as bone was demineralized, the mineral maturity (or mineralization index) was not measured, and we have shown that a great difference in the ratio could be due to the region of bone analyzed (the ratio double in old bone compared new bone). Finally, Paschalis et al. have used UV photolysis on cross-links peptide (1 night and 3 days), and analyzed their peptide before and after UV irradiation [7]. They showed that a destruction of PYD by UV photolysis after 1 night (60%) lead to a decrease of only 4% in the 1660 cm^−1^ peak area. After total destruction (3 days), the authors mentioned that “no amide I spectral feature near 1660 cm^−1^ was observed”. However, in amide I vibration on the spectra, a peak near 1655 cm^−1^ is present, suggesting that the UV photolysis shift the peak but do not suppress it. This shift can be simply due to a modification of secondary structure of collagen upon UV photolysis, due to the increase of temperature under UV. Indeed, we observed that if the UV bath was not refreshed, the temperature dramatically increased, and this could lead to a collagen denaturation. To avoid this phenomenon, we have performed this experiment on bovine bone (2 different ages) at 4°C on all the duration of the experiment.

Our study has a limitation. The 1660/1690 cm^−1^ area ratio was measured on bone slides, allowing to obtain a spatial distribution of collagen maturity and the quality of the mineral phase. However, the quantification of ECL by HPLC was performed on whole bone analysis.

In summary, no discrimination in the 1660/1690 cm^−1^ area ratio was observed between the control and lathyritic bones, whereas a great decrease in PYD content were measured in lathyritic bones. These observations were confirmed by an independent laboratory on large areas by chemometric analysis. Moreover, no correlation was found between the 1660/1690 cm^−1^ ratio and the PYD/DHLNL ratio in bovine bone. However, the 1660/1690 cm^−1^ area ratio was correlated to the mineral maturity and mineralization index, suggesting that it likely reflects the change in secondary structure of collagen in relation with mineralization process rather than the modification of the enzymatic cross-links. Thus, the term “collagen maturity” needs to be used when the 1660/1690 cm^−1^ area ratio is measured instead of the term “cross-links ratios”.
